# Extending Healthy Ageing Narratives in Sub-Saharan Africa: Expert Viewpoint

**DOI:** 10.3390/healthcare14010088

**Published:** 2025-12-30

**Authors:** Daniel Katey, Senyo Zanu, Abigail Agyekum, Anthony Kwame Morgan

**Affiliations:** 1 M.A. Program in Interdisciplinary Aging Studies, Trent University, 1600 West Bank Drive, Peterborough, ON K9L 0G2, Canada; 2 Trent Centre for Aging & Society, Trent University, 1600 West Bank Drive, Peterborough, ON K9L 0G2, Canada; 3 Department of Natural and Built Environment, Sheffield Hallam University, City Campus, Sheffield S1 1WB, UK; senyozanu069@gmail.com; 4 Laurel Dene Care Home, Care UK, 117 Hampton Road, Hampton Hill, Hampton TW12 1JQ, UK; abigailagyekum339@gmail.com; 5 Department of Applied Social Sciences, The Hong Kong Polytechnic University, Kowloon, Hong Kong; anthoniomorgano280@gmail.com

**Keywords:** age-friendly environments, healthy ageing, Sub-Saharan Africa, healthcare accessibility, older adults

## Abstract

The nexus of rapid demographic transition and underdeveloped geriatric infrastructure poses a critical, yet understudied challenge in Sub-Saharan Africa (SSA). As global life expectancies rise, SSA’s older population is projected to triple by 2050, intensifying the need for sustainable age-friendly environments (AFEs) and robust healthy ageing interventions. Informal or family caregiving structures, while vital, are under strain from rapid urbanisation and shifting social dynamics, creating a compelling gap between need and provision. This expert viewpoint draws on the authors’ professional and scholarly experience regarding population ageing, AFEs, and healthy ageing to provide a comprehensive outlook on these issues in SSA. Selective literature searches were conducted in Google Scholar, Scopus and PubMed using targeted keywords and MESH terms, including “ageing in Africa”, “ageing in Sub-Saharan Africa”, “healthy ageing in Africa”, “healthy ageing in Sub-Saharan Africa”, “population ageing in Africa”, “population ageing in Sub-Saharan Africa”, “age-friendly environment in Africa”, and “age-friendly environment in Sub-Saharan Africa.” The authors argue that rapid population ageing in SSA is outpacing existing informal care arrangements, necessitating a strategic shift towards the development of age-friendly environments and more coordinated healthy ageing interventions to bridge the widening gap between demographic change and geriatric support systems. This paper underscores the necessity of proactive, evidence-based policy implementation to secure the well-being of SSA’s burgeoning older population.

## 1. Overview

The creation of age-friendly environments (AFEs) is becoming increasingly important as the world undergoes rapid demographic changes and urbanisation [1,2]. Evidence suggests that people are living longer in the twenty-first century than ever before due to improved living standards, better nutrition and sanitation, and advances in healthcare [3]. The increase in life expectancy globally has brought into focus the concept of healthy ageing, which refers to the process of developing and maintaining the functional ability that enables well-being in old age [4]. As life expectancy increases, ensuring that longer lives are also healthier and more fulfilling becomes crucial [5,6]. Healthy ageing, therefore, involves maintaining physical, mental, and social well-being even in old age, supported by access to healthcare, opportunities for social engagement, and support for maintaining independence [1,7]. Creating AFEs is essential in this endeavour, as it helps support the functional abilities of older adults and enhances their quality of life. This paper focuses on the intersection of the themes of AFEs and healthy ageing within the context of Sub-Saharan Africa (SSA), exploring the current state and future directions for fostering healthy ageing in the subregion.

## 2. Background

The ageing population faces myriad challenges, including age-related diseases, social isolation, lack of access to healthcare, and spatial accessibility issues [8]. Evidence suggests that to support and enable healthy ageing, there is a need to create AFEs that consider the complex dynamics of ageing and the ageing process across varied socio-spatial contexts [9,10]. The concept of AFEs refers to physical and social settings that accommodate the needs of older adults, making it easier for them to navigate, access services, and participate in community life [11]. These environments include accessible public spaces, transportation, housing and healthcare services specifically designed to support people as they age [11]. Such environments, according to Garner and Holland [12], also foster social inclusion, safety and respect for older people. The World Health Organization (WHO) [4] notes that AFEs support healthy ageing by increasing and preserving intrinsic capacity across the life-course and enabling higher functional ability. The WHO’s Age-friendly Cities and Communities program, therefore, seeks to encourage cities and communities to assess and enhance their physical and social environments to better meet the needs of the ageing population [13]. In that regard, Quia et al. [14] described an age-friendly environment as one that is inclusive, accessible and promotes healthy ageing.

AFEs play an important role in defining the quality of health and welfare of older people [4,15]. Thus, older adults’ feelings of place attachment and psychosocial health are heavily influenced by their views of neighbourhood safety and social cohesion [16]. Studies conducted in the United States and Canada (two of the most important countries in the age-friendly conversation) suggest that neighbourhood housing and services are strong determinants of health and well-being at old age [17]. Meanwhile, evidence suggests that some age-friendly initiatives may be limited in their capacity to achieve larger-scale results aimed at addressing broader ageing problems [18]. This calls for a comprehensive approach targeted at ensuring the sustainability of age-friendly initiatives across diverse spatial contexts and older adult groups [19].

Focusing on SSA is critical due to the region’s rapid demographic shift towards an ageing population [20], coupled with unique challenges such as higher poverty rates [21], limited healthcare accessibility [4] and weaker social protection systems [22,23]. The health disparities faced by older adults in SSA [24], along with the lack of comprehensive policies addressing their needs [25], further underscore the importance of this paper. Moreover, the cultural context of SSA, where traditional values and family structures significantly influence geriatric care, also necessitates a tailored examination of age-friendly initiatives [26]. By highlighting these issues, our paper aims to contribute to a more inclusive understanding of ageing in SSA and provide evidence-based recommendations for regional development.

Moreover, understanding the intersection of AFEs and healthy ageing in SSA is crucial for several reasons. For instance, for policy, this understanding offers insights that can guide the development of targeted interventions to address the specific needs of older adults in the region. For research, it identifies gaps in the current literature and suggests areas for further investigation. More so, for practice, the paper provides practical suggestions for national governments, NGOs, healthcare providers, development partners and organisations working to improve the health and well-being of older adults on the subcontinent. Thus, this paper seeks to create awareness of some important sustainable development strategies that can promote healthy ageing and create supportive environments for SSA’s ageing population.

## 3. Methodology

This expert viewpoint presents knowledge and interpretive judgement of the authors on the broader themes of AFEs, healthy ageing and population ageing to provide an outlook on the nexus in SSA. It is intended to influence debate, guide practice, or highlight gaps, rather than present raw data [27]. Literature searches were conducted in Google Scholar, Scopus, and PubMed using keywords and MESH terms including: “ageing in Africa”, “ageing in Sub-Saharan Africa”, “healthy ageing in Africa”, “healthy ageing in Sub-Saharan Africa”, “population ageing in Africa”, “population ageing in Sub-Saharan Africa”, age-friendly environment in Africa”, and “age-friendly environment in Sub-Saharan Africa.” After searching, we selectively synthesised the most relevant studies to frame the discussion on healthy ageing in SSA. However, the evidence cited is selective, not exhaustive, as the methodology is interpretive and argumentative, not systematic. The paper’s evidence was not collected systematically; instead, sources were based on their relevance to current practice, debates and their alignment with the authors’ normative judgements [27]. Thus, the process involved critical reflection on existing knowledge, gaps in empirical research, and integration of policy consensus. The strength lies in contextual insight and professional consensus, not in reproducibility [28,29]. The expert viewpoint was employed because evidence is scarce and emerging, thereby enriching discussions on healthy ageing narratives in SSA.

## 4. Evidence and Critical Reflections

### 4.1. Population Ageing, Healthy Ageing, and AFEs in SSA

SSA’s ageing population is projected to triple from 53 million in 2009 to 150 million by 2050 [30], with an increasing number of older adults living in urban areas [20]. Despite growing concerns about developing inclusive and resilient spaces, discussions about urban development in the context of ageing are relatively sparse [31]. Recent studies highlight the necessity of addressing these issues. For instance, research conducted in Nigeria emphasises the importance of ensuring older adults have access to social and physical environments that promote physical activity in their communities [32]. A study conducted in Cameroon reported that older people face significant challenges in accessing affordable housing in urban areas, including Bamenda [1]. These challenges reportedly stemmed from the unavailability of accessible sidewalks, lack of public restrooms, scarcity of elevators and escalators in public facilities and the absence of reserved parking spaces for older adults and individuals with disabilities [1]. Moreover, poor healthcare systems, inadequate health infrastructure and substandard healthcare delivery continue to pose significant threats to age-friendly healthcare initiatives on the subcontinent. In the city of Conakry in Guinea, evidence suggests that many public buildings are not age-friendly due to narrow stairways and the general lack of elevators [33]. This evidence underscores the urgent need for comprehensive urban planning and development policies that cater to the needs of the ageing population in individual SSA countries.

### 4.2. Socio-Cultural Context of SSA and Healthy Ageing

In SSA, traditional values and family structures play a profound role in shaping geriatric care practices and perceptions of ageing [26]. The concept of respect for older persons is deeply rooted in many African cultures, where older adults are often revered as repositories of wisdom and custodians of cultural heritage [26,34]. Family networks traditionally assume responsibility for the care and support of older family members, reflecting communal values of reciprocity and intergenerational solidarity [26]. This familial caregiving model extends beyond immediate relatives to include extended family and community networks, ensuring that older adults are integrated into social networks and receive practical assistance as they age [34]. However, rapid urbanisation, economic shifts and changing social dynamics are increasingly impacting entrenched traditional caregiving arrangements [35]. For instance, rural-urban migration and labour mobility often separate younger family members from older relatives, weakening daily caregiving support and reducing co-residence. At the same time, economic pressures, changing gender roles and the monetisation of care are straining extended family systems, leading to greater caregiving burdens on fewer individuals and increasing the risk of unmet care needs among older adults. Furthermore, older adults are often marginalised and discriminated against in the provision of essential public services, including geriatric healthcare [36,37]. This discrimination extends to social protection schemes, where older adults are overlooked in policy-making [37,38]. The widespread ageism in these societies adversely affects the mental and physical well-being of older adults and hinders their ability to contribute effectively to their communities [39]. Studies have shown that ageism can lead to social isolation, reduced access to medical care and economic insecurity, exacerbating the vulnerabilities faced by older adults [38,40].

### 4.3. Healthy Ageing and AFEs Intersection in SSA

Healthy ageing and AFEs intersection refers to the interplay between promoting health and well-being among older adults and creating environments that support their needs and dignity [41,42]. This concept encompasses the integration of policies, services and infrastructure designed to enhance the quality of life of older people [41,43]. Despite the increasing recognition of the importance of healthy ageing globally, evidence suggests that there is a diminished concern among national governments, non-governmental bodies, and other key stakeholders in SSA for prioritising the health and well-being of the ageing population within the broader national development agenda [15,44]. This lack of prioritisation is often attributed to the pervasive political and socio-economic instability in many SSA countries [45]. State funds are frequently redirected towards addressing political disputes and providing “fundamental” social amenities, leaving limited resources for other developmental initiatives that could support the ageing population [45]. Additionally, entrenched issues, including corruption, favouritism, unsustainable state projects and poor international development deals, continue to hinder developmental progress [46,47]. A review of national constitutions across some SSA countries reveals a significant marginalisation of older adults in national development planning initiatives [39]. These systemic issues pose major threats to achieving the goals of the United Nations Decade of Healthy Ageing (2021–2030), which seeks to improve the lives of older people, their families, and the communities in which they age [48]. Thus, without targeted interventions, the ageing population in SSA will continue to face challenges that compromise their quality of life, including limited access to healthcare and inadequate social services [49]. Addressing these problems is therefore crucial for fostering inclusive and sustainable development. By integrating age-friendly policies and promoting healthy ageing initiatives, SSA can create environments that support the well-being of older adults and enhance their ability to contribute to societal advancement.

## 5. Practical and Policy Considerations

### 5.1. Prioritising the Needs of Older People in National Development Plans

Governments and policymakers in SSA need to prioritise the needs of older adults in their national development plans. One critical step is the establishment of dedicated departments or agencies responsible for ensuring that policies and programmes across all sectors systematically address the needs of older people. Such institutions should coordinate and oversee interventions that promote ageing within the community, ensuring that older adults receive sustained policy attention and support. In Nigeria, for example, the celebration of the ‘International Day of Older Persons Against Ageism’ promotes intergenerational initiatives and raises awareness about the needs and benefits of ageing in society [50]. During these engagements, older adults share lived experiences of ageing, often drawing on culturally resonant proverbs that challenge entrenched ageist ideologies. These engagements reposition older adults as active contributors to society, strengthen intergenerational dialogue and promote more positive social attitudes towards ageing. Embedding such perspectives within national development frameworks can enhance policy responsiveness, reduce age-based discrimination and ensure that ageing-related priorities are meaningfully integrated into broader development agendas across SSA.

Beyond policy formulation, effective responses to population ageing require strong multisectoral collaboration. Governments should therefore work closely with Non-Governmental Organisations (NGOs), local assemblies and other relevant institutions to address the complex social, cultural and health-related needs of older adults. Through joint initiatives such as public awareness campaigns and community-based programmes, these partnerships can deepen public understanding of the challenges faced by older people and encourage their active participation in society. For instance, Nigeria’s Help Unite Great Generations in Nigeria (HUGGING) programme organises popular artist performances, radio and television programmes, and drama workshops to raise awareness and amplify the voices of older adults [51]. Such collaborations help reduce social isolation and ageism, while ensuring that older adults remain visible, valued and engaged within their communities [52].

However, recognising the complexity and multidimensional nature of the challenges of population ageing across the SSA region, governments should consider tailoring these recommendations to suit their country-specific needs, resources, and governmental structures. For example, in Ghana, the establishment of the Center for Ageing Studies at the University of Ghana provides a model for advancing multidisciplinary research and innovation in ageing within a local context [53]. Such institutions can support the development of context-specific strategies by generating local evidence, building capacity, and informing programmes that respond to country-specific ageing-related needs and resources. In contrast, a country like Kenya, with its unique socio-political landscape, might focus on integrating ageing policies within its existing social protection programmes, such as the Older Persons Cash Transfer Program, which provides direct financial support to older adults [54]. This programme helps to alleviate poverty among older adults and can be expanded or adapted to include other supportive services tailored to the Kenyan context. In South Africa, where there is a relatively more robust healthcare infrastructure, efforts might focus on enhancing geriatric healthcare services and integrating AFEs designs into urban planning. The country’s National Development Plan 2030 highlights the importance of creating inclusive urban environments that cater to the needs of all age groups [55].

Given that traditional values and family structures continue to play a central role in supporting older adults in SSA, governments should prioritise policies that strengthen intergenerational solidarity, encourage family caregiving, and integrate cultural practices into ageing programmes. Evidence from Asian contexts, such as South Korea’s community-based housing and Singapore’s family-inclusive ageing initiatives, suggests that aligning ageing policies with prevailing cultural norms can enhance public acceptance and long-term sustainability. SSA countries can adapt similar models by promoting family support networks, community dialogues, and culturally sensitive awareness campaigns, ensuring ageing strategies align with local traditions and social realities. By doing so, SSA countries can more effectively address the diverse challenges of population ageing and improve the quality of life for their ageing populations.

### 5.2. Supporting Ageing in Place Through Age-Friendly Housing and Infrastructure

Governments ought to collaborate with city authorities, planners, estate developers, and construction companies to create age-friendly physical environments that enable older adults to live independently, safely, and with dignity. In line with the World Health Organization’s Age-Friendly Cities and Communities framework—particularly the domains of outdoor spaces and buildings and housing—such collaborations should prioritise the design and implementation of accessible and inclusive infrastructure for older adults. They should prioritise features like step-free entrances, wider doors, elevators, accessible toilet facilities, well-maintained sidewalks, and safe, walkable neighbourhoods. The development and preservation of green spaces and parks are also essential for promoting mobility, social interaction, and well-being among older adults. These physical features are critical for reducing fall risks, improving accessibility, and supporting ageing in place. International examples demonstrate the feasibility and benefits of these approaches. In Singapore, government investment in age-friendly housing has ensured accessibility through thoughtful architectural designs [56]. Moreover, in towns with a higher proportion of older adults, infrastructure is retrofitted to AFEs. This includes installing handrails and levelling uneven roads, allowing older people to go about their day independently and safely [56]. In Seoul, the government implemented the Borin Housing projects, which aimed to design barrier-free houses for older adults [56]. Accordingly, automatic sliding doors, elevators and handrails were all installed in these facilities while floors of all shared spaces and common areas were kept at even heights. These were done to facilitate mobility among older adults accessing the facilities [56]. Given that both South Korea and African countries share some socio-cultural and political commonalities, SSA countries need to consider adopting or adapting this approach. For instance, both South Korea and most African countries share several socioeconomic commonalities, including post-colonial development [57], rapid urbanisation [57], and challenges of inequality and poverty [58,59]. Therefore, SSA governments should designate funds towards developing age-friendly physical infrastructure, providing subsidies and tax waivers to reliable estate developers and property owners to prioritise age-friendly housing. Evidence suggests that community design, housing, transportation, and community engagement can have a positive impact on geriatric health, well-being, and the ability to age in place [60,61,62]. However, entrenched socio-economic constraints, particularly widespread poverty, may limit the capacity of some African governments to implement these interventions at scale. Governments should therefore seek international development support, foster public–private partnerships and support community-led initiatives to expand the provision of age-friendly physical environments across the region.

### 5.3. Improved Healthcare Accessibility and Affordability

Finally, one of the crucial components of creating an age-friendly environment is to make healthcare accessible and affordable to the ageing population [63]. An age-friendly environment supports older adults in maintaining their health, independence, and quality of life [64]. This can be achieved through policies such as subsidising medical costs [6], expanding health insurance coverage [65], and increasing the number of healthcare facilities and professionals trained in geriatric care [66]. Governments can increase the availability of healthcare services for older people by developing specialised healthcare services, including geriatric care units in hospitals [66]. For instance, in Ghana, the National Health Insurance Scheme (NHIS), established in 2007, exempts individuals aged 70 years and older (or 60 if they were Social Security and National Insurance Trust [SSNIT] contributors) from paying the annual premium subscription fees required to access the health insurance benefits [67,68]. This initiative has increased the utilisation of formal healthcare services among older people [67]. Furthermore, a Fellowship in Geriatric Medicine was initiated by the Ghana College of Physicians and Surgeons as part of efforts to prepare the health service for an ageing population [69]. Through this initiative, evidence suggests that geriatric clinics have been established in major hospitals across the country, as well as in some community hospitals [52]. These examples demonstrate that with appropriate policies and targeted programmes, other SSA countries can improve healthcare accessibility and affordability for the ageing population, thereby fostering an age-friendly environment that supports healthy ageing.

## 6. Strengths, Limitations and Future Studies

This viewpoint offers an attempt to consolidate perspectives on healthy ageing in SSA. A notable strength is its integration of global guidance, such as WHO frameworks, with the demographic and health system realities of SSA, thereby providing contextually relevant insights. The interpretive nature of the synthesis allows for nuanced reflection on policy and practice gaps, particularly in workforce development and culturally tailored interventions. Limitations arise from the absence of a systematic review methodology, which restricts comprehensiveness and introduces potential bias through selective evidence use. Future studies should extend this line of inquiry by employing systematic and longitudinal approaches, conducting cross-country comparisons, and generating empirical data to strengthen the evidence base. Researchers are encouraged to explore innovative models of community-based care and curriculum reform, ensuring that ageing in SSA is addressed with scientific rigour, cultural sensitivity, and sustainable policy alignment.

## 7. Conclusions

This paper highlights the critical need for AFEs and healthy ageing initiatives in SSA. As the region experiences a rapid demographic shift towards an ageing population, it faces unique challenges such as limited healthcare access and weaker social protection systems. These entrenched issues highlight the need for comprehensive policies and programmes that prioritise the well-being of older adults. Moreover, creating AFEs requires a multifaceted approach that includes enhancing healthcare accessibility and affordability, fostering multi-sectoral collaborations, and promoting intergenerational programmes. By drawing on successful examples from countries like Ghana, Nigeria, Singapore, and South Korea, SSA countries can implement strategies tailored to their specific socio-economic contexts. Initiatives such as subsidising medical costs, expanding health insurance coverage, and establishing geriatric care units have proven effective in improving the quality of life for older adults. Furthermore, the cultural context of SSA, where traditional values and family structures significantly influence geriatric care, necessitates a tailored examination of age-friendly initiatives. All in all, addressing the health disparities and marginalisation faced by older adults in SSA will improve their well-being and contribute to the region’s development and stability. Our paper, therefore, provides evidence-based recommendations that can guide policymakers in enhancing quality of life for older people in SSA, ultimately contributing to a more equitable and sustainable future for all generations.

## Data Availability

Data sharing does not apply to this article as no new data were created or analysed in the study.
